# Characterization of Electronic Cigarette Aerosol and Its Induction of Oxidative Stress Response in Oral Keratinocytes

**DOI:** 10.1371/journal.pone.0154447

**Published:** 2016-05-25

**Authors:** Eoon Hye Ji, Bingbing Sun, Tongke Zhao, Shi Shu, Chong Hyun Chang, Diana Messadi, Tian Xia, Yifang Zhu, Shen Hu

**Affiliations:** 1 School of Dentistry, University of California Los Angeles, Los Angeles, United States of America; 2 School of Medicine, Division of Nanomedcine, University of California Los Angeles, Los Angeles, United States of America; 3 California Nanosystems Institute, University of California Los Angeles, Los Angeles, United States of America; 4 Department of Environmental Health Sciences, University of California Los Angeles, Los Angeles, United States of America; 5 Peking University, School of Physics, Beijing, China; Queen Mary University of London, UNITED KINGDOM

## Abstract

In this study, we have generated and characterized Electronic Cigarette (EC) aerosols using a combination of advanced technologies. In the gas phase, the particle number concentration (PNC) of EC aerosols was found to be positively correlated with puff duration whereas the PNC and size distribution may vary with different flavors and nicotine strength. In the liquid phase (water or cell culture media), the size of EC nanoparticles appeared to be significantly larger than those in the gas phase, which might be due to aggregation of nanoparticles in the liquid phase. By using *in vitro* high-throughput cytotoxicity assays, we have demonstrated that EC aerosols significantly decrease intracellular levels of glutathione in NHOKs in a dose-dependent fashion resulting in cytotoxicity. These findings suggest that EC aerosols cause cytotoxicity to oral epithelial cells *in vitro*, and the underlying molecular mechanisms may be or at least partially due to oxidative stress induced by toxic substances (e.g., nanoparticles and chemicals) present in EC aerosols.

## Introduction

Electronic cigarettes (ECs) are battery-operated devices for a user to inhale an aerosol rather than cigarette smoke. ECs typically have a heating element that generates aerosols by atomizing a liquid solution known as E-liquid, which usually contain a mixture of selected level of nicotine, propylene glycol or glycerin as solvent, and flavors additives. Awareness and use of ECs have greatly increased in the past few years, particularly among young people and women [1]. Although ECs have been proposed as long-term substitutes for traditional smoking or as a tool for smoking cessation, scarce experimental data are available on their safety and health related risks [2]. Most of the current studies are focused on understanding EC users’ behavior or pathological symptoms through the use of approaches including questionnaires compiled by the EC users, surveys from online forums or systematic review of published literature [2–4]. While studies have suggested that the use of ECs substantially decreased cigarette consumption without causing significant side effects in smokers not intending to quit [5], others emphasized that the health effects caused by EC use are not well understood and there is a wide range of reported positive and negative health effects. While the use of the EC may help reduce the number of cigarettes smoked and withdrawal symptoms, the effects are mainly related to a short period of use, and data on long-term efficacy and safety of ECs is currently lacking, which will be of utmost importance to form the basis for guidelines and regulatory decisions on ECs [3, 4].

While the effects of conventional cigarette smoke on human health have been well documented through *in vitro* and *in vivo* model studies, little direct work has been done to understand the health risks of ECs, particularly those on the oral cavity. A recent study on E-liquids demonstrated that menthol additives of E-liquid show a harmful effect on human periodontal ligament fibroblasts. The incubation with menthol-flavored E-liquids led to a significant reduction of cell proliferation and viability, which might indicate that menthol additives should be avoided for ECs [6]. Although E-liquid itself is not the same as EC aerosol generated after heating, this study highlights the importance of investigating oral health-related effects of ECs. So far, studies of ECs at molecular levels have been performed on lung/airway epithelia cells. E-liquid has been found to increase inflammation and virus infection in primary human airway epithelial cells [7]. Exposure to ECs impairs pulmonary anti-bacterial and anti-viral defenses in a mouse model, and vapors produced by ECs and E-liquids with flavorings induce toxicity, oxidative stress, and inflammatory response in lung epithelial cells and in mouse lung [8, 9]. Nevertheless, to the best of our knowledge, there is essentially no data describing the effects of EC aerosols on oral epithelial cell function and mechanisms involved in inducing the effects.

In this study, we have generated and characterized EC nanoparticles in gas and liquid phases using a combination of advanced technologies. By using *in vitro* high-throughput cytotoxicity assays and quantitative PCR (qPCR), we have further demonstrated that EC aerosols significantly decrease intracellular levels of glutathione (GSH) in NHOKs in a dose-dependent fashion, induce the expression of heme oxygenase 1 (HO-1) and cause cytotoxicity to normal oral epithelial cells. This suggests that EC aerosols may induce oxidative stress response and toxicological outcomes in the oral cavity.

## Materials and Methods

### Cell culture

Normal human oral keratinocytes (NHOKs) were maintained in EpiLife culture media supplemented with the human keratinocyte growth supplement (Invitrogen, Carlsbad, CA, USA), as described previously [10]. The cell line was provided by Dr. Wei Chen at the University of California, Los Angeles. NHOKs were treated with the EC aerosol-impinged EpiLife culture media for 24 hours prior to harvesting of cells for the analysis. Generation of EC aerosols and EC aerosol-impinged cultured media are described below in details.

### Generation of EC aerosols

E-liquid with different nicotine strength and flavors were used to generate EC aerosols. A homemade puffing machine composed of a compressed air source, a solenoid valve, and a Raspberry Pi (Raspberry Pi Foundation, UK), which serves as a timer and solenoid valve controller, was used to puff the ECs by pushing clean air through the EC from the front air hole. A piece of Python code running on the Raspberry Pi, which can be adjusted by changing the code, accurately controlled the puff duration and puff interval. The flow rate of the inlet air was calibrated by a flow meter DC-Lite (Drycal, Bios Inc., US). Particle number concentration (PNC) and size distribution of EC aerosols were measured as a function of puff duration from approximately 2 to 5 seconds inside a 320 L stainless-steel chamber. The chamber was tightly closed to avoid air exchange with ambient air. During the experimental period, the relative humidity and temperature inside the chamber were controlled at 30 ± 10% and 24 ± 1°C, respectively. Details of the experiment set up can be found elsewhere [11].

### Measurement of particle number concentration and size distribution

The particle number concentration and size distribution of EC aerosols were measured by a Condensation Particle Counter (CPC 3785, TSI Inc., Shoreview, MN) and an Scanning Mobility Particle Sizer (SMPS 3080, TSI Inc., Shoreview, MN), respectively. The sampling flow rate of the SMPS was 0.6 L/min and the measurement range was 7–289 nm (100 s up scan, 20 s down scan). The SMPS starts to work right after each puff. The particle measurements were all repeated five times for each puff duration. After each measurement, the chamber was flushed by clean air until the total particle number concentration in the chamber was less than 1000 cm^-3^.

### Physicochemical characterization of EC particles in liquid

For the liquid phase particle size distribution, the EC aerosols were impinged into liquid (water or culture media) for 10 min, by using the same puffing system at the same flow rate, 1 L/min. High throughput dynamic light scattering (HT-DLS, Dynapro™ Plate Reader, Wyatt Technology) was performed to determine the particle size and size distribution of the EC aerosols in water and cell culture media. Transmission electron microscopy (TEM), using a JEOL 1200 EX (accelerating voltage 80 kV), was used to observe the morphology and to determine the primary size of EC aerosol nanoparticles. Elemental analysis of the EC aerosols were determined by energy-dispersive X-ray spectroscopy (EDX) using a FEI Titan 80/300 microscope. Quantitative elemental analysis of the EC aerosols was determined by inductively coupled plasma optical emission spectrometry (ICP-OES). 500 μL of the impinged EC sample was digested by 3 mL of concentrated nitric acid at 90°C for 3 h. The digested solution was dried by evaporation at 120°C, and 8 mL of 5% nitric acid was added for ICP-OES measurement.

### Cytotoxicity assays

The cytotoxicity of EC aerosols in NHOK cells was determined by a ATP assay using the ATPliteTM firstep (Perkin-Elmer, Boston, MA) [12]. After 24 h exposure to EC aerosol-impinged culture medium in a 96-well plate, the culture medium was removed, and cells were washed three times with PBS and incubated with 100 μL of reconstituted ATPlite firstep reagent for 10 min. The luminescence intensity was recorded on a SpectraMax M5 microplate spectrophotometer (Molecular Devices, Sunnyvale, CA).

### Determination of intracellular GSH

A GSH-Glo assay kit (Promega, Madison, WI) was used to determine the intracellular GSH levels after EC aerosol exposure in NHOKs [13]. The NHOKs were exposed to EC aerosol particles in a 96-well plate at 37°C and 5% CO_2_ for 24 h. After exposure, the cellular supernatant was removed and 100 μL of GSH-Glo reaction buffer containing Luciferin-NT and glutathione S-transferase was added to each well in the plate and incubated at room temperature with constant shaking for 30 min. Then, 100 uL of Luciferin D detection reagent was added to each well and the plate was incubated at room temperature with constant shaking for another 15 min. The luminescent signal was quantified using a SpectraMax M5 microplate reader (Molecular Devices; Sunnyvale, CA).

### Quantitative real time PCR

Total RNA was isolated from cultured cells using the RNeasy Mini Kit (Qiagen, Valencia, CA). With Superscript II reverse transcriptase (Invitrogen, #18064–022), 1.5μg of RNA per sample was converted into cDNA. The primers sequences were: HO-1-F, CAGGCAGAGAATGCTGAGTT; HO-1-R, GCTTCACATAGCGCTGCA; NRF-2-F, CGGTATGCAACAGGACATTG; NRF-2-R, ACTGGTTGGGGTCTTCTGTG; β-actin-F, GCGCGGCTACAGCTTCA; β-actin-R, CTTAATGTCACGCACGATTTCC. To quantify HO-1 and NRF-2 gene expression levels in untreated and EC aerosol-treated NHOK samples, 1μl of diluted cDNA solution (1:10) was mixed with primers, nucleotides and the SYBR Green I MasterMix (Roche, Indianapolis, IN) in a 96 well PCR plate and the reaction was performed on a CFX96 qPCR system (Bio-Rad, Hercules, CA).

### Statistical analysis

Statistical significance was determined by two-tailed Student's t-test for two-group analysis. For all the Figs, the values shown represent mean ± SEM.

## Results

### Puff duration and particle number concentration (PNC) of EC aerosols

**Fig 1:**
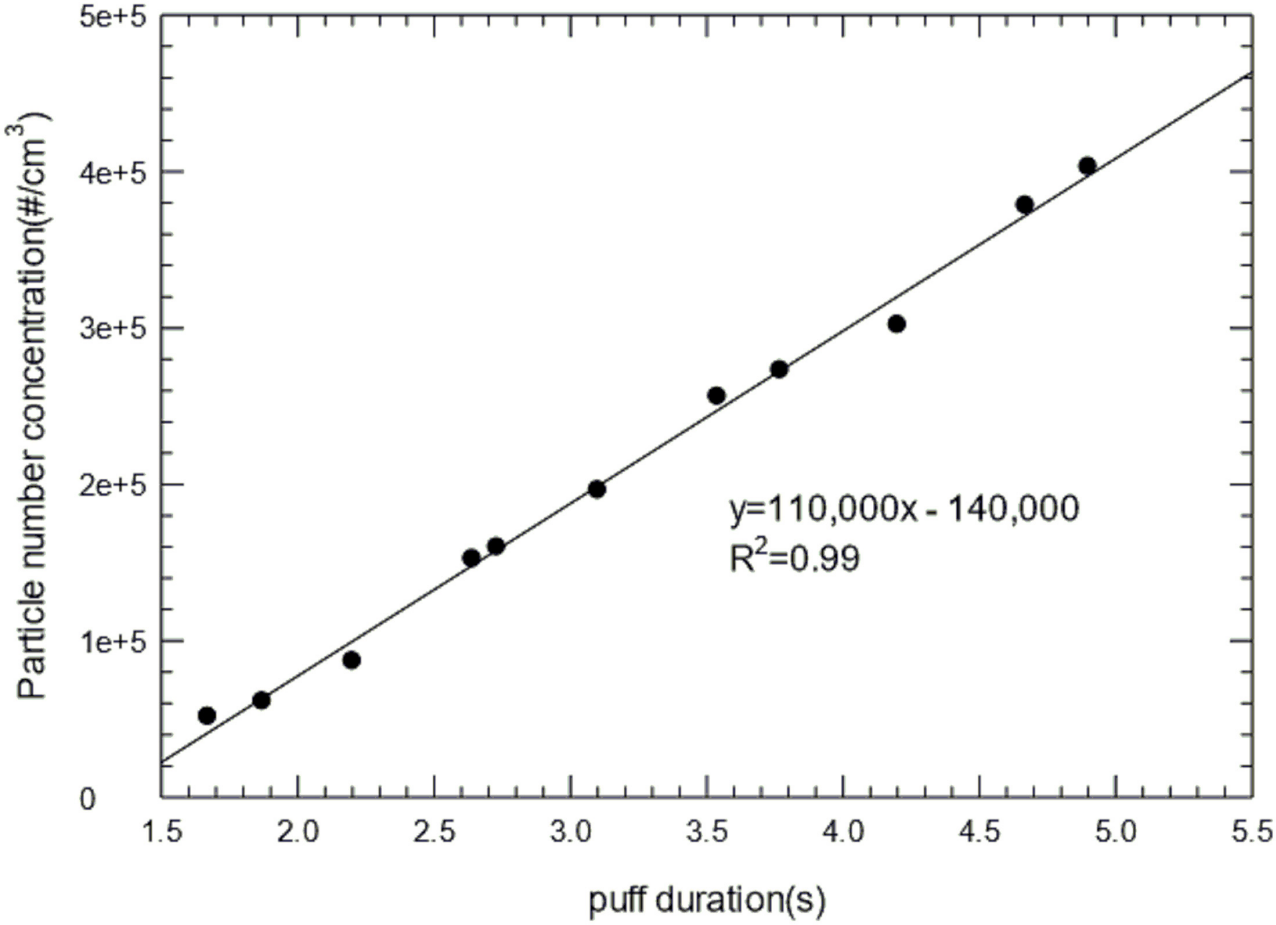
(a) Particle number concentration (PNC) as a function the puff duration. Particle number was determined by Condensation Particle Counter (CPC).

We have generated EC aerosols and measured PNC and size distribution as a function of puff duration from approximately 2 to 5 seconds. The results are presented in Fig 1, which shows a strong positive correlation (R^2^ = 0.99) between puff duration and PNC.

### PNC and size distribution of EC aerosols at different flavors and nicotine strength

**Fig 2:**
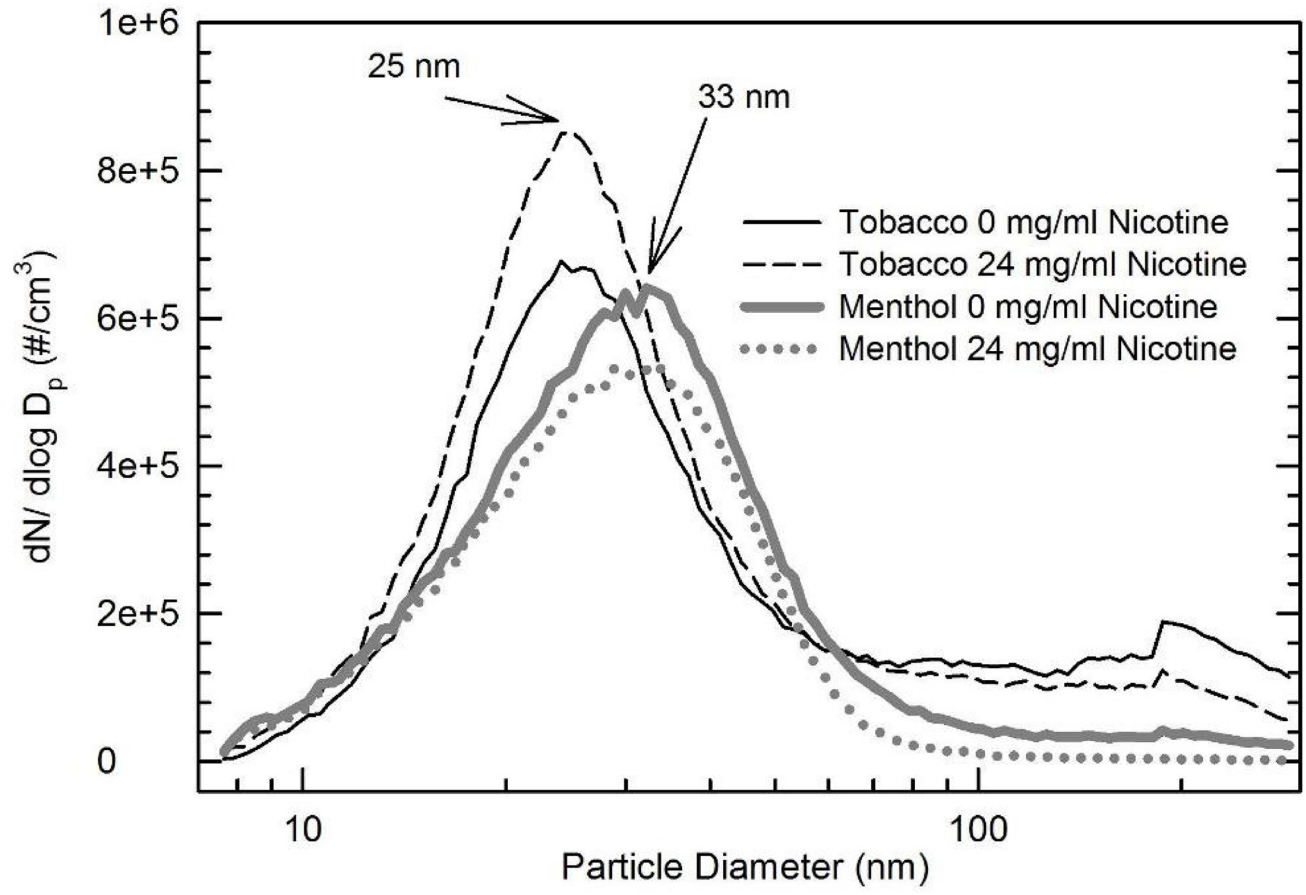
Comparison of particle size distribution of aerosol emissions from tobacco (0 and 24 mg/ml nicotine) and menthol (0 and 24 mg/ml nicotine) flavored ECs.

The PNC and size distribution of EC aerosols of different flavors and nicotine strength were also compared (Fig 2). The menthol-flavored EC generates particles with larger sizes (33 nm) compared to the tobacco flavors (25 nm). It also appears to have generated fewer nanoparticles than the tobacco flavored by 13–35%. Compared to zero nicotine, at 24 mg/ml nicotine level, the tobacco flavored showed 9% increase in PNC, but the menthol flavored had approximately 20% decrease in PNC.

### Characterization of EC nanoparticles in liquid phase

**Fig 3:**
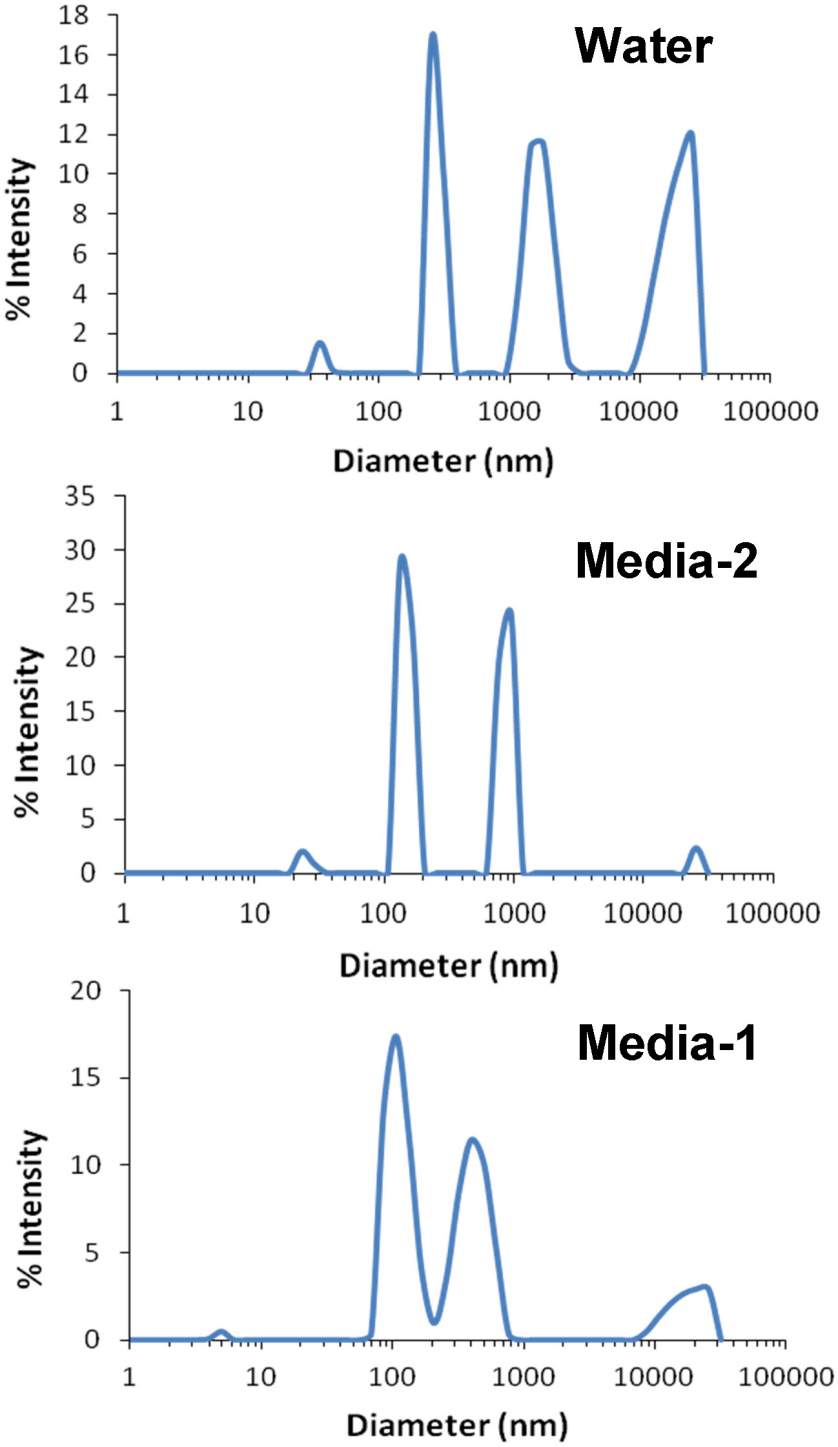
Hydrodynamic diameters of EC aerosols in different dispersing media (water or cell culture media). Cell media 1: DMEM with fetal bovine serum. Cell media 2: EpiLife media with growth supplement. Hydrodynamic size of EC aerosols was determined using high throughput dynamic light scattering (HT-DLS).

**Fig 4:**
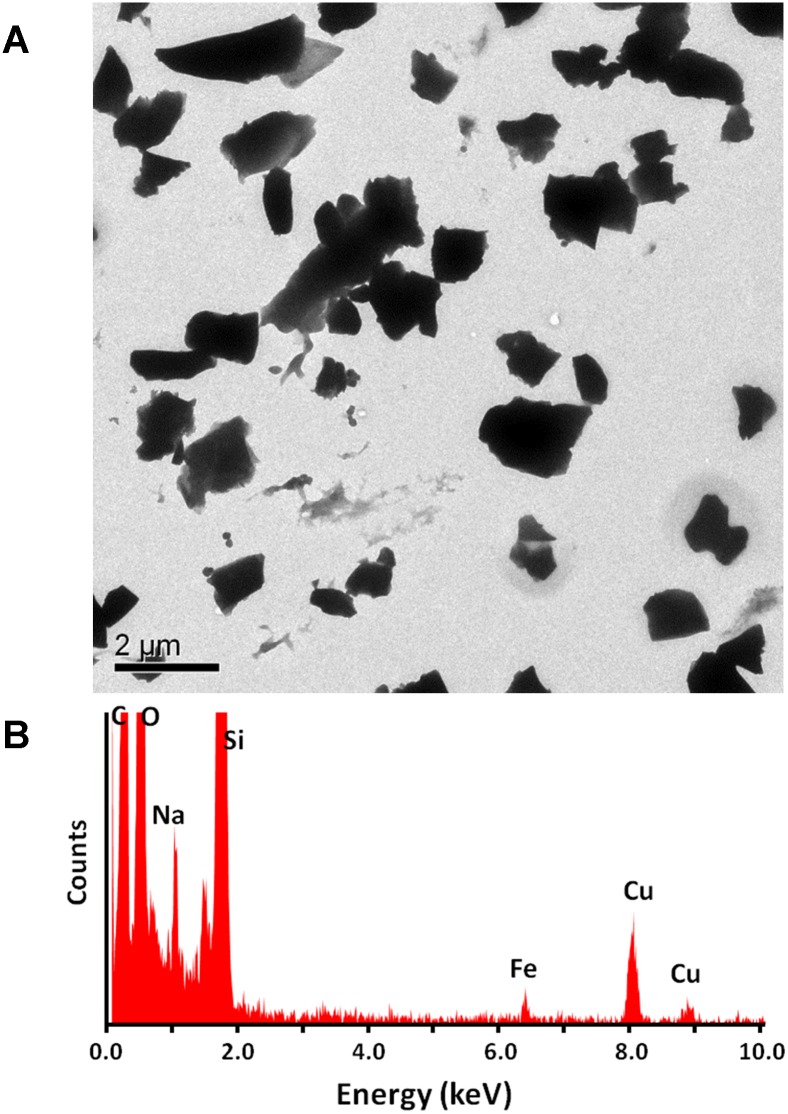
Characterization of EC aerosols impinged in water. (A) TEM analysis of EC aerosol nanoparticles in water. (B) EDX analysis of EC aerosols that identified elemental composition of EC nanoparticles.

To perform in vitro cellular studies with NHOKs, the EC aerosol was impinged into the culture medium for NHOK (KGM medium with human keratinocyte growth supplement) for 10 min. Since the suspension of the aerosol particles in aqueous solution will likely change their physicochemical properties, we compared the hydrodynamic size distribution of EC aerosols in NHOK culture medium, DMEM medium and water (different dispersion media) using high-throughput dynamic light scattering analysis (DLS) (Fig 3). Our data indicate that when the EC particles were impinged in water, their hydrodynamic size is around 1181.1±340.1 nm. In contrast, when they were prepared in NHOK culture media, their hydrodynamic size is significantly reduced to 442.3 nm (Table 1), which reflects the dispersion effect of proteins including growth factors that are present in the culture media as a result of the formation of a protein corona on the particle surface that provides electrostatic hindrance preventing agglomeration. When the EC particles were impinged in DMEM medium containing fetal bovine serum, the hydrodynamic size is further reduced to 328.5±9.1 nm, due to dispersal effect of fetal bovine serum as a result of the formation of a protein corona on the particle surface, which contributes to the suspension stability [14]. Fig 4A shows the transmission electron microscope (TEM) image, revealing the flake-like morphology of EC aerosol nanoparticles in the water. Furthermore, energy-dispersive X-ray spectroscopy (EDX) was used to determine the elemental components of EC aerosols (Fig 4B). Silicon (Si), iron (Fe), and sodium (Na) were found in the EC particles. Copper (Cu) was also found but it came from the TEM grid, not the nanoparticles. Quantitative assessment of EC aerosols by inductively coupled plasma optical emission spectrometry (ICP-OES) further confirms the EDX analysis. Besides the elements identified by EDX, ICP-OES also shows the existence of calcium (Ca), magnesium (Mg), and sulfur (S) (Table 2).

**Table 1:** Hydrodynamic diameter of EC aerosols in different dispersing media analyzed by DLS.

Sample	Hydrodynamic diameter (nm)
EC in water	1181.1 ± 340.1
EC in culture media-1	442.3 ± 125.2
EC in culture media-2	328.5 ± 9.1

Note: Culture media-1: EpiLife with growth supplement for NHOKs. Culture media-2: DMEM with fetal bovine serum

**Table 2:** Elemental analysis of EC aerosols by ICP-OES.

Element	Concentration (mg/L)
Ca	0.121±0.001
Fe	0.828±0.005
Mg	0.042±0.000
Na	2.289±0.081
S	0.764±0.003
Si	0.117±0.002

### EC aerosol-induced oxidative stress response and cytotoxicity in oral epithelial cells

**Fig 5:**
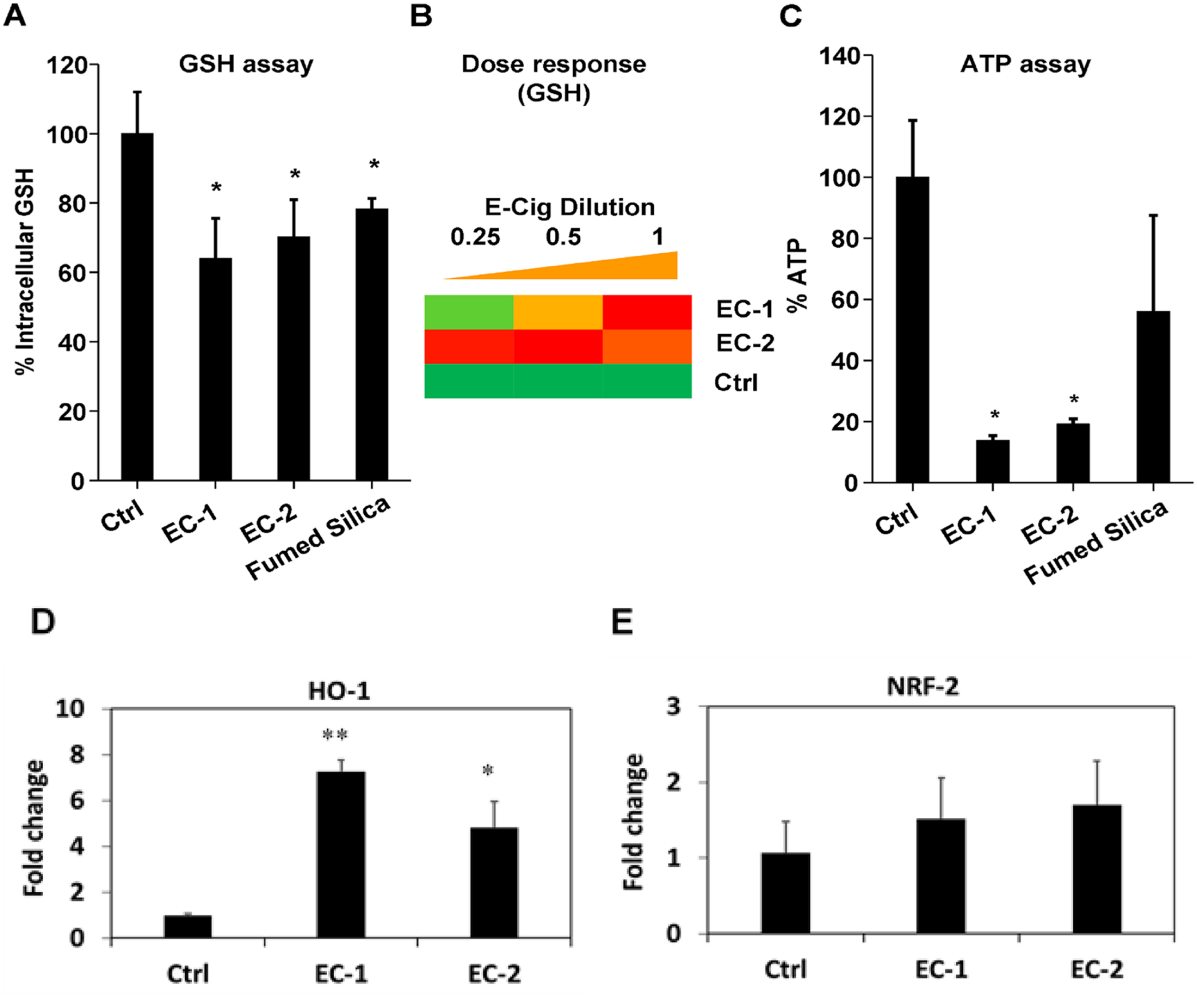
Oxidative stress and cytotoxicity induced by EC aerosols in NHOKs. (A) Intracellular GSH levels in NHOKs after exposure to EC aerosols. NHOKs were exposed to EC aerosols for 24 h and intracellular GSH levels were determined using a GSH-Glo assay. Fumed silica (100 μg/ml) was used as a positive control. *p<0.05 compared to untreated control cells. (B) Heat maps to show the dose-dependent increase in oxidative stress induced by EC in NHOKs. Conditions are the same as (A). (C) Cell viability of NHOKs after exposure to EC aerosols for 24 h was determined using ATP assay. The cell viability of the EC-treated cells was normalized to the value of non-treated control cells, for which the viability was regarded as 100%. Fumed silica (100 μg/ml) was used as a positive control. *p<0.05 compared to untreated control cells. (D&E) qPCR analysis of heme oxygenase 1 (HO-1) and nuclear factor (erythroid-derived 2)-like 2 (NRF-2) expression in NHOKs after exposure to EC aerosols. *p<0.05; ** p<0.01 compared to untreated control cells.

In vitro analysis of EC aerosol-treated NHOKs show that EC aerosols are capable of inducing oxidative stress as indicated by significant decrease of intracellular glutathione (GSH) levels (Fig 5A). Similar to previously published data [15], the use of fumed silica as a positive control demonstrated a significant decrease in cellular GSH in NHOKs. GSH level decrease is also dose-dependent (Fig 5B). Oxidative stress represents a dynamic equilibrium between antioxidant defense that acts to restore redox equilibrium and oxidant injury responses that can result in toxicological outcomes. We found the injurious oxidative stress in NHOKs leads to significant cytotoxicity as indicated by the ATP assay (Fig 5C). As shown in Fig 5D, qPCR analysis demonstrated that EC aerosols induced the expression of HO-1 in NHOKs, but NRF-2 expression was not significantly altered (Fig 5E).

## Discussion

EC creates aerosols that consist of nanoparticles and contain small amount of chemicals that may cause toxicological outcome to human oral cavity. Smoking characteristics such as puffing topography or EC device voltage and physicochemical characteristics of vaporized nicotine and other chemical products in ECs are profoundly different when compared to conventional cigarettes. How these toxic substances/nanoparticles from EC aerosols and related smoking/physicochemical characteristics affect the oral cavity remains largely unknown. Considering the increasing popularity of ECs in the general population, there is an urgent need to characterize EC aerosols and assess their biological hazard on oral epithelial cells.

In this study, an impinging method was chosen because it minimizes EC aerosols loss due to evaporation, which is inevitable if filter collection method is used [16, 17]. In addition, impinging method allows EC aerosols to be directly captured in the medium and avoids intermediate steps such as extraction, which are necessary if a filter trapping is used.

Previous studies have shown significant amounts of nanoparticles are present in EC aerosols [16, 18–20]. These observed PNC in gas phase ranged from 1.8×10^9^ cm^-3^ to 8.4×10^9^ cm^-3^ and count median diameter (CMD) ranged from 14 nm to 458 nm. Puffing topography and device voltage of ECs have been found to affect EC aerosol characteristics. Fuoco *et al*. reported that longer puff duration was associated with higher PNC [20]. This has been confirmed by our studies (Fig 1). Ohta *et al*. [21] measured carbonyls emitted from ECs at voltages from 1.5 V to 7.5 V and found increased carbonyl levels when the voltage was above 3 V. In addition, our studies indicate that the PNC and size distribution of EC aerosol emissions may vary with different flavors and nicotine strength (Fig 2). The menthol flavored EC generated particles with larger sizes compared to the tobacco flavors. It also produced fewer nanoparticles than the tobacco flavored EC. Nicotine levels appeared to affect the PNC differently from the menthol flavor. Compared to zero nicotine, the tobacco flavored EC showed a 9% increase in PNC at 24 mg/ml nicotine level, while the menthol flavored EC had approximately 20% decrease in PNC. In contrast, Fuoco and colleagues reported that flavors did not significantly change the PNC [22]. However, they did find that the PNC significantly increased when nicotine levels in E-liquid are higher, which agrees with our measurements for the tobacco flavor. These finding demonstrates the complexity and knowledge gap in EC aerosol characteristics and highlights the importance to systematically evaluate the impacts of puff duration, EC device voltage and E-liquid composition on EC aerosol characteristics. This information might be important to link the physicochemical properties of EC aerosols under controlled conditions to the biological/toxicological outcomes to the oral cavity.

We also measured the size of EC aerosol nanoparticles in liquid phase. EC aerosols were impinged into water or the cell culture medium for NHOKs and the nanoparticles present in the culture medium were measured with TEM. The EC nanoparticles in liquid phase appeared to be significantly larger than those in the gas phase (Fig 3), and this might be due to aggregation of nanoparticles in the liquid phase. The difference in EC aerosol size distribution and aggregation status between the air and aqueous solution needs to be investigated because it may generate differential toxicological outcomes to cells.

Small trace amount of toxic chemicals and metals were found in the E-liquid and EC aerosols in previously reported studies. For example, formaldehyde, which is assumed to be the product of thermal dehydration of the glycerin or glycols, was detected in EC aerosols [23–25]. However, another study suggested that the formaldehyde may come from exhaled breath rather than from the ECs [16]. Diethylene and ethylene glycol were detected as impurities in E-liquid [26] and in EC aerosols [27]. However, the prevalence of these impurities in E-liquid remains unclear. Kim and Shin [28] found substantial amounts of tobacco specific nitrosamines (TSNA) which are carcinogenic, while McAuley and colleagues [27] detected that TSNA in EC aerosols were at least six times lower than in tobacco smoke. In addition, heavy metal nanoparticles (i.e. Sn, Ag, Fe, Ni, Al, Cr) were found in EC aerosols which could be resulted from the oxidation of the heating coil [29]. We also examined the elemental components of the EC nanoparticles in the liquid phase and Fe, Si and Na were detected with the EDX analysis. Due to their unique physicochemical properties including high surface area, metal/metal oxide nanoparticles exhibit higher dissolution rate or surface reactivity compared to their bulk form. Our previous studies revealed that metal/metal oxide nanoparticles could cause oxidative stress and cytotoxicity *in vitro* and acute lung inflammation *in vivo* [12, 30]. The exact role of particles in EC-induced cytotoxicity needs to be determined in future studies.

There is a practical challenge when studying the physicochemical characteristics of EC aerosols. Due to the chemical complexity of EC aerosols, it appears to be very difficult to link a specific chemical component of EC aerosols to the toxicological outcomes. As reported earlier, cigarette smoke is a complex mixture consisting of more than 5600 identified chemical constituents of which approximately 150 have been identified so far as “tobacco smoke toxicants” [31, 32]. This can be partially solved by including well-characterized controls (*e*.*g*., metal/metal oxide nanoparticles) [12]. Trace metals or chemicals identified by other instrumental analysis methods (e.g., ICP or LC/GC-MS) would provide useful information on the potential toxic components in EC aerosols, and by including reference standards for *in vitro* studies, we might be able to identify major toxic components in EC aerosols.

More importantly, our data suggest that EC aerosols may cause cytotoxicity to human oral keratinocytes via oxidative stress response. High-throughput cytotoxicity assays confirmed that EC aerosols are capable of inducing oxidative stress in NHOKs as indicated by significant dose-dependent decrease of intracellular GSH levels. We also found the injurious oxidative stress causes cytotoxicity of NHOKs as indicated by the ATP assay. In addition, similar to our previous studies on the toxicology of nanoparticles [12, 33], HO-1 expression was found to be induced in NHOKs by EC aerosols, which correlate well with the high-throughput cytoassay data on decreased intracellular GSH level and cytotoxicity.

As a summary, we have prepared and measured PNC and size distribution of EC aerosols in the gas phase and found that there is a strong positive correlation between puff duration and PNC. We have also characterized the hydrodynamic size of EC nanoparticles in liquid phase (water and cell cultured media) and determined their elemental composition. Because PNC, particle size and chemical content are highly relevant to the toxicity of EC aerosols, it is important to characterize these physiochemical parameters and understand the effect of smoking characteristics such as puff duration and device voltage on these physiochemical parameters. Our *in vitro* assays have shown that EC aerosols could cause oxidative stress responses and induce cytotoxicity in oral epithelial cells. These data suggest that EC aerosols damage human keratinocytes *in vitro*, and the underlying molecular mechanisms may be or at least partially due to oxidative stress and inflammation responses induced by toxic substances (e.g., nanoparticles and chemicals) present in EC aerosols. Therefore, further safety assessment of toxicological and pathological effects of EC aerosols on human health is certainly important. In the future, our research focus is to investigate pathological effects and oxidative stress/inflammation responses in the animal models caused by EC aerosol exposure.
