# Correction: Characterization of Electronic Cigarette Aerosol and Its Induction of Oxidative Stress Response in Oral Keratinocytes

**DOI:** 10.1371/journal.pone.0169380

**Published:** 2016-12-29

**Authors:** Eoon Hye Ji, Bingbing Sun, Tongke Zhao, Shi Shu, Chong Hyun Chang, Diana Messadi, Tian Xia, Yifang Zhu, Shen Hu

There is an error in reference 11. The correct reference is: Zhao T, Shu S, Guo Q, Zhu Y. Effects of design parameters and puff topography on heating coil temperature and mainstream aerosols in electronic cigarettes. Atmospheric Environment. 2016; 134: 61–69. doi: 10.1016/j.atmosenv.2016.03.027.
